# Association Between Colorectal Cancer Screening and Survival in Patients Older Than 70 Years: Results of A National Multicenter Retrospective Study

**DOI:** 10.1002/jso.70206

**Published:** 2026-02-22

**Authors:** Matteo Rottoli, Giacomo Calini, Giovanni Castagna, Alice Gori, Stefano Cardelli, Antonino Spinelli, Gianluca Pellino, Alessandro Bianconi, Matteo Fiore, Riccardo Rosati, Mario Morino, Nicolò de Manzini, Andrea Pietrabissa, Luigi Boni, Gilberto Poggioli, COVID‐CRC Study Group, Angela Romano, Angela Belvedere, Antonio Lanci Lanci, Daniele Parlanti, Gabriele Vago, Anna Paola Pezzuto, Anna Canavese, Gerti Dajti, Stefano Cardelli, Caterina Catalioto, Iris S. Russo, Daniele Morezzi, Ludovica Maurino, Eleonora Filippone, Dajana Cuicchi, Elio Jovine, Raffaele Lombardi, Chiara Cipressi, Maria F. Offi, Cristina Larotonda, Silvana B. Puglisi, Augusto Barbosa, Roberto Vaiana, Paolo M. Bianchi, Carlo Tonti, Claudio Codignola, Luigi Zorcolo, Angelo Restivo, Simona Deidda, Marcello E. Marchetti, Luca Ippolito, Gaya Spolverato, Salvatore Pucciarelli, Francesco Marchegiani, Giacomo Ghio, Gaia Zagolin, Dajana Glavas, Monica Tomassi, Ugo Elmore, Lorenzo Gozzini, Riccardo Calef, Francesco Puccetti, Andrea Cossu, Andrea Vignali, Marco E. Allaix, Gaspare Cannata, Erica Lombardi, Carlo A. Ammirati, Chiara Piceni, Piero Buccianti, Riccardo Balestri, Marco Puccini, Daniele Pezzati, Roberto d'Ischia, Vito F. Asta, Benedetta Sargenti, Giacomo Taddei, Federica Bonari, Giulia Boni, Alessandro Ferrero, Michela Mineccia, Federica Gonella, Marco Palisi, Francesco Danese, Valeria Cherubini, Serena Perotti, Michele Carvello, Fabio Carbone, Antonio Luberto, Eleonora Calafiore, Francesca De Lucia, Matteo Sacchi, Diego Sasia, Maria C. Giuffrida, Edoardo Ballauri, Mathieu Cardile, Serena Armentano, Elsa Beltrami, Gabriele Preve, Barbara Vercellone, Marta Mozzon, Cristina Folliero, Chiara Lirusso, Massimo Vecchiato, Antonio Ziccarelli, Davide Gattesco, Luisa Moretti, Sara Crestale, Filippo Banchini, Patrizio Capelli, Andrea Romboli, Gerardo Palmieri, Luigi Conti, Nicholas Rizzi, Deborah Bonfili, Paola Germani, Edoardo Osenda, Sara Cortinovis, Carlotta Giunta, Stefano Fracon, Hussein Abdallah, Selene Bogoni, Nazario Portolani, Riccardo Nascimbeni, Sarah Molfino, Guido A. M. Tiberio, Ilenia Garosio, Giulia Lamperti, Diego Rigosa, Giorgio Ercolani, Leonardo Solaini, Davide Cavaliere, Andrea Avanzolini, Fabrizio D'Acapito, Leonardo L. Chiarella, Daniela Di Pietrantonio, Domenico Annunziata, Roberta Piccolo, Mario Sorrentino, Mauro Pansini, Alessandro Cojutti, Michele Graziano, Francesco Callegari, Laura Balzarotti, Vitale R. Dameno, Antonio Cattaneo, Giuliano Santolamazza, Caterina Altieri, Riccardo Magarini, Tommaso Dominioni, Luigi Pugliese, Andrea Peri, Marta Botti, Benedetta Sargenti, Francesco Salvetti, Elisa Cassinotti, Ludovica Baldari, Valentina Messina, Vera D'Abrosca, Pasquale Cianci, Rocco Tumolo, Domenico Gattulli, Enrico Restini, Marina Minafra, Maria Grazia Sederino, Bernardino Bottalico, Pierluigi Pilati, Boris Franzato, Genny Mattara, Ottavia De Simoni, Andrea Barina, Marco Tonello, Andrea Muratore, Marcello Calabrò, Nicoletta S. Federico Pipitone, Bruno Cuzzola, Elena Herranz Van Nood, Mariangela Azzellino, Nicola Passuello, Alvise Frasson, Enzo Mammano, Luca Faccio, Fabrizio Vittadello, Alice Bressan, Giacomo Sarzo, Nicolò Tamini, Massimo Oldani, Luca Cigagna, Francesca Carissimi, Giulia De Carlo, Edoardo Baccalini, Luca Nespoli, Alessio Giordano, Stefano Cantafio, Lucrezia Grifoni, Davide Matani, Serena Livi, Daniele Delogu, Fabrizio Scognamillo, Antonio Marrosu, Luca Guerrini, Giampaolo Ugolini, Federico Ghignone, Giacomo Frascaroli, Nicola Albertini, Davide Zattoni, Giovanni Taffurelli, Isacco Montroni, Francesco Colombo, Piergiorgio Danelli, Andrea Bondurri, Anna Maffioli, Alessandro Bonomi, Isabella Pezzoli, Francesco Cammarata, Orlando Goletti, Mattia Molteni, Alberto Assisi, Giorgio Quartierini, Corrado Da Lio, Daunia Verdi, Isabella Mondi, Claudia Peluso, Lorenzo Macchi, Marta Tanzanu, Federico Zanzi, Sara Pellegrini, Jacopo Andreuccetti, Rossella D'Alessio, Giusto Pignata, Michele De Capua, Ilaria Canfora, Luca Ottaviani, Pasquale Lepiane, Andrea Balla, Antonio De Carlo, Federica Saraceno, Rosa Scaramuzzo, Anna Guida, Daniele Aguzzi, Paolo Bellora, Sergio Gentilli, Manuela Monni, Herald Nikaj, Nicola Cillara, Alessandro Cannavera, Antonello Deserra, Carla Margiani, Roberta Cabula, Manuela Dettori, Giulia Gramignano, Giovanni Lezoche, Monica Ortenzi, Elena S. Orlandoni, Federica Curzi, Francesca Vitali, Perla Capomagi, Miriam Palmieri, Mario Giuffrida, Paolo Del Rio, Elena Bonati, Tommaso Loderer, Federico Cozzani, Matteo Rossini, Stefano Agnesi, Gabriella T. Capolupo, Marco Caricato, Filippo Carannante, Gianluca Mascianà, Martina Marrelli, Valentina Miacci, Sara Lauricella, Valeria Tonini, Maurizio Cervellera, Salvatore Pisconti, Concetta Lozito, Juliana Shahu, Claudia Mongelli, Giulia Morelli, Lodovico Sartarelli, Giuseppe S. Sica, Leandro Siragusa, Giulia Bagaglini, Andrea M. Guida, Marzia Franceschilli, Vittoria Bellato, Cristina Fiorani, Antonio Taddei, Matteo Risaliti, Ilenia Bartolini, Maria N. Ringressi, Luca Tirloni, Letizia Laface, Emmanuele Abate, Massimiliano Casati, Pietro Gobbi, Enrico Opocher, Nicolò M. Mariani, Andrea Pisani Ceretti, Marco Giovenzana, Beatrice Giuliani, Martina Sironi, Ugo Grossi, Giacomo Zanus, Giulio Aniello Santoro, Marco Brizzolari, Eugenio De Leo, Simone Novello, Krizia Aquilino, Francesco Milardi, Stefano Olmi, Matteo Uccelli, Marta Bonaldi, Giovanni C. Cesana, Marco Bindi, Raffaele Galleano, Antonio Langone, Massimiliano Botto, Angelo Franceschi, Elena Gambino, Maurizio Ronconi, Silvia Casiraghi, Giovanni Casole, Salvatore L. Ciulla, Giovanni Terrosu, Sergio Calandra, Edoardo Scarpa, Vittorio Cherchi, Giacomo Calini, Lisa Martinuzzo, Lucrezia Clocchiatti, Davide Muschitiello, Andrea Romanzi, Barbara Vignati, Alberto Vannelli, Roberta Scolaro, Maria Milanesi, Fabrizio Rossi, Giuseppe Canonico, Alessandro Anastasi, Tommaso Nelli, Marco Barlettai, Riccardo Fratarcangeli, Carmela Di Martino, Andrea Damigella, Elvira Adinolfi, Arianna Birindelli, Lucio Taglietti, Sara E. Dester, Francesco Fleres, Eugenio Cucinotta, Francesca Viscosi, Santino A. Biondo, Giorgio Badessi, Nivia Catarsini, Carmelo Mazzeo, Daniela Rega, Paolo Delrio, Carmela Cervone, Alessia Aversano, Silvia De Franciscis, Massimiliano Di Marzo, Bruno Marra, Ugo Pace, Antonio Amato, Paola Batistotti, Elisa Mina, Alberto Serventi, Pierfrancesco Lapolla, Andrea Mingoli, Paolo Sapienza, Gioia Brachini, Bruno Cirillo, Enrico Fiori, Daniele Crocetti, Ilaria Clementi, Gennaro Martines, Arcangelo Picciariello, Giovanni Tomasicchio, Rigers Dibra, Giuseppe Trigiante, Marcella Rinaldi, Giuliano Lantone, Alberto Porcu, Teresa Perra, Antonio M. Scanu, Claudio F. Feo, Alessandro Fancellu, Maria L. Cossu, Giorgio C. Ginesu, Alberto Patriti, Diego Coletta, Filippo Petrelli, Paola A. Greco, Claudia Spadoni, Giovanna Cassiani, Federica Bianchini, Marco Arganini, Matteo Bianchini, Bruno Perotti, Matteo Palmeri, Stefano Scabini, Selene Deiana, Giacomo Carganico, Davide Pertile, Domenico Soriero, Emanuela Fioravanti, Beatrice Sperotto, Bruno Nardo, Daniele Paglione, Veronica Crocco, Marco Doni, Mariasara Osso, Roberto Perri, Gianluca M. Sampietro, Carlo Corbellini, Leonardo Lorusso, Carlo A. Manzo, Maria Cigognini, Caterina Baldi, Giuseppe Palomba, Giovanni Aprea, Marianna Capuano, Raffaele Basile, Roberta Tutino, Marco Massani, Laura Marinelli, Nicola Canitano, Tiziana Pilia, Mauro Podda, Adolfo Pisanu, Valentina Murzi, Silvia Incani, Federica Frongia, Giuseppe Esposito, Gaetano Luglio, Francesca P. Tropeano, Gianluca Pagano, Eduardo Spina, Giuseppe De Simone, Michele Cricrì, Fausto Catena, Carlo Vallicelli, Nicola Zanini, Diana Ronconi, Francesco Favi, Carlo Mazzucchelli, Girolamo Convertini, Leonardo Vincenti, Valeria Andriola, Cinzia Bizzoca, Carlo V. Feo, Nicolò Fabbri, Marta Fazzin, Antonio Pesce, Silvia Gennari, Marco Torchiaro, Silvia Severi, Alice Frontali, Greta Bracchetti, Stefano Granieri, Christian Cotsoglou, Massimo Carlini, Giorgio Lisi, Domenico Spoletini, Maria R. Mastrangeli, Michela Campanelli, Michele Manigrasso, Marco Milone, Giovanni D. De Palma, Sara Vertaldi, Alessia Chini, Francesco Maione, Alessandra Marello, Francesco Selvaggi, Guido Sciaudone, Lucio Selvaggi, Francesco Menegon Tasselli, Giacomo Fuschillo, Lidia Oddis, Simona Grande, Michele Grande, Simona Ascanelli, Laura Chimisso, Filippo Aisoni, Eleonora Rossin, Francesco Pepe, Francesco Marchetti, Biagio Picardi, Stefano Rossi, Simone Rossi Del Monte, Matteo Picarelli, Irnerio A. Muttillo, Carlo Ratto, Angelo A. Marra, Angelo Parello, Francesco Litta, Paola Campennì, Veronica De Simone, Francesco Pata, Cristiana Riboni, Emanuele Rausa, Valerio Celentano, Tommaso Violante

**Affiliations:** ^1^ Surgery of the Alimentary Tract IRCCS Azienda Ospedaliero‐Universitaria di Bologna Bologna Italy; ^2^ Department of Medical and Surgical Sciences Alma Mater Studiorum University of Bologna Bologna Italy; ^3^ Department of Biomedical Sciences Humanitas University Milan Italy; ^4^ IRCCS Humanitas Research Hospital Milan Italy; ^5^ Department of Advanced Medical and Surgical Sciences Università degli Studi della Campania Luigi Vanvitelli Naples Italy; ^6^ Colorectal Surgery University Hospital Vall d'Hebron Barcelona Spain; ^7^ Gastrointestinal Surgery Division IRCCS San Raffaele Hospital Milan Italy; ^8^ AOU Città della Salute e della Scienza Turin Italy; ^9^ General Surgery Department University Hospital of Trieste Trieste Italy; ^10^ Department of Surgery University of Pavia and Fondazione IRCCS Policlinico San Matteo Pavia Italy; ^11^ Department of General and Minimally Invasive Surgery, Fondazione IRCCS ‐ Ca' Granda ‐ Ospedale Maggiore Policlinico di Milano University of Milan Milan Italy; ^12^ Chirurgia A e d'Urgenza IRCCS AOU c/o OM, IRCCS Azienda Ospedaliero‐Universitaria di Bologna Bologna Italy; ^13^ Fondazione Poliambulanza Brescia Brescia Italy; ^14^ Unità operativa di Chirurgia Coloproctologica ‐ AOU Cagliari Cagliari Italy; ^15^ Department of Surgical Oncological and Gastroenterological Sciences University of Padova ‐ General Surgery 3, Azienda Ospedale Università di Padova Padova Italy; ^16^ Azienda Ospedaliero‐Universitaria Pisana Pisa Italy; ^17^ Azienda Sanitaria Ospedaliera Ordine Mauriziano Umberto I° Torino Italy; ^18^ Santa Croce and Carle Hospital Cuneo Italy; ^19^ Chirurgia Generale, Azienda ospedaliero‐universitaria S. Maria della Misericordia Udine‐ASUFC Udine Italy; ^20^ UO Chirurgia Generale Vascolare di Piacenza Piacenza Italy; ^21^ Dipartimento di Chirurgia Università degli Studi di Parma Parma Italy; ^22^ U.O. Chirurgia Generale 3 ‐ ASST Spedali Civili Brescia, Università di Brescia Brescia Italy; ^23^ Chirurgia generale e TOA, Ospedale Morgagni‐Pierantoni Forlì Italy; ^24^ U.O. Chirurgia Generale Ospedale di Latisana‐Palmanova, Azienda Ospedaliera Universitaria Friuli Centrale Italy; ^25^ Ospedale civile “G. Fornaroli” Magenta Italy; ^26^ SC Chirurgia Generale e Mini‐invasiva, Fondazione IRCCS Ca' Granda Ospedale Maggiore Policlinico ‐ Milano Italy; ^27^ UOC Chirurgia Generale, Ospedale Lorenzo Bonomo Andria Italy; ^28^ Unit of Surgical Oncology of Digestive Tract Veneto Institute of Oncology IOV‐IRCCS Padova Italy; ^29^ Chirurgia Generale Ospedale E. Agnelli Pinerolo Italy; ^30^ U.O.C. Chirurgia Generale OSA, io DIDAS Chirurgia, Azienda Ospedale‐Università Padova Padova Italy; ^31^ UO Chirurgia 1, Fondazione IRCCS San Gerardo dei Tintori, Università di Milano‐Bicocca Monza Italy; ^32^ UO di Chirurgia Generale, Nuovo Ospedale “S.Stefano”, Azienda ASL Toscana Centro Italy; ^33^ Patologia Chirurgica AOU Sassari Italy; ^34^ UO Chirurgia Generale di Ravenna‐Faenza, AUSL Romagna Italy; ^35^ Division of General Surgery ‐ L. Sacco University Hospital‐ Milano Italy; ^36^ Chirurgia Generale Humanitas Gavazzeni Bergamo Italy; ^37^ Department of General Surgery Mirano Hospital Venice Italy; ^38^ Chirurgia d'Urgenza, Santa Maria delle Croci ‐ Ravenna Italy; ^39^ General Surgery 2, ASST Spedali Civili of Brescia Italy; ^40^ Ospedale San Paolo Civitavecchia Roma Italy; ^41^ Clinica Chirurgica Ospedale Maggiore della Carità ‐ Novara Italy; ^42^ UOC Chirurgia Generale PO Santissima Trinità ASL Cagliari Cagliari Italy; ^43^ Oncologia Medica, PO Businco, ARNAS Cagliari Cagliari Italy; ^44^ SSD Oncologia, PO Nostra Signora di Bonaria San Gavino, ASL Medio Campidano Italy; ^45^ Clinica di Chirurgia Generale e d'urgenza, Ancona Torrette Italy; ^46^ Clinica Chirurgica Generale ‐ AOU Parma Italy; ^47^ UOC Chirurgia colorettale, Fondazione Policlinico Campus Bio Medico Roma Italy; ^48^ Ospedale Santissima Annunziata Taranto Italy; ^49^ Policlinico Tor Vergata Roma Italy; ^50^ Azienda Ospedaliero Universitaria Careggi Firenze Italy; ^51^ Ospedale Vittorio Emanuele III Italy; ^52^ ASST Santi Paolo e Carlo Milano Italy; ^53^ II Surgery Unit Regional Hospital Treviso, DISCOG, University of Padua Italy; ^54^ Policlinico San Marco GSD Zingonia Italy; ^55^ Ospedale San Paolo Savona Italy; ^56^ Ospedale di Gardone V.T. ‐ ASST Spedali Civili di Brescia Brescia Italy; ^57^ Clinica Chirurgica, Azienda Sanitaria Universitaria Friuli Centrale ASUFC Udine Italy; ^58^ Department of General Surgery Valduce Hospital Como Italy; ^59^ Ospedale San Giovanni di Dio Firenze Italy; ^60^ UOC Chirurgia ‐ Ospedale di Esine (BS) ‐ ASST Valcamonica Italy; ^61^ AOU G Martino Policlinico di Messina, Department of General and Emergency Surgery Italy; ^62^ Colorectal Surgical Oncology, Department of Abdominal Oncology Istituto Nazionale Tumori‐IRCCS “Fondazione G. Pascale” Naples Italy; ^63^ SC Chirurgia Generale Imperia Imperia Italy; ^64^ Policlinico Umberto I Sapienza Università di Roma Roma Italy; ^65^ Chirurgia Generale "M.Rubino" Azienda Ospedaliero Universitaria Policlinico Bari Italy; ^66^ Azienda Ospedaliero Universitaria di Sassari Italia; ^67^ AO Ospedali Riuniti Marche Nord Italy; ^68^ Ospedale Unico della Versilia ‐ Azienda Usl Toscana Nord‐ovest Italy; ^69^ Unità Operativa Chirurgia Generale ad Indirizzo Oncologico ‐ IRCCS Ospedale Policlinico San Martino Genova Italy; ^70^ U.O.C. di Chirurgia Generale "Falcone" ‐ Azienda Ospedaliera di Cosenza ‐ Università della Calabria Cosenza Italy; ^71^ Division of Surgery Rho Memorial Hospital ‐ ASST Rhodense ‐ Rho Milan Italy; ^72^ AOU Federico II di Napoli ‐ UOC chirurgia endoscopica Napoli Italy; ^73^ Chirurgia 1 ‐ Azienda ULSS2 Marca Trevigiana ‐ Ospedale Regionale di Treviso Italy; ^74^ Policlinico di Monserrato, Chirurgia d'urgenza Cagliari Italy; ^75^ Azienda Ospedaliera Universitaria Federico II Napoli Italy; ^76^ Chirurgia Generale e d'Urgenza, Ospedale Bufalini di Cesena, AUSL della Romagna Cesena Italy; ^77^ Chirurgia Generale Ospedaliera, Policlinico di Bari Italy; ^78^ Azienda Unità Sanitaria Locale di Ferrara, Università di Ferrara Italy; ^79^ General Surgery Unit, ASST Vimercate Vimercate Italy; ^80^ UOC Chirurgia Generale, Ospedale Sant'Eugenio Roma Italia; ^81^ Department of Clinical Medicine and Surgery “Federico II” University of Naples Naples Italy; ^82^ Università della Campania Luigi Vanvitelli Napoli Italy; ^83^ UOSD Chirurgia d'urgenza Tor Vergata Roma Italy; ^84^ UO Chirurgia 2 Azienda Ospedaliero‐Universitaria Ferrara Ferrara Italy; ^85^ Chirurgia Generale e d'Urgenza Ospedale San Filippo Neri ASL Roma 1 Rome Italy; ^86^ Fondazione Policlinico Universitario Agostino Gemelli, IRCCS Rome Italy; ^87^ Department of Surgery Nicola Giannettasio Hospital Corigliano‐Rossano Italy; ^88^ La Sapienza University Rome Italy; ^89^ EOC Ospedale Regionale di Lugano Lugano Switzerland; ^90^ Unit of Hereditary Digestive Tumors Fondazione IRCCS‐National Cancer Institute Milan Italy; ^91^ Chelsea and Westminster Hospital NHS Foundation Trust London UK; ^92^ Department of Surgery and Cancer Imperial College London UK

**Keywords:** colorectal cancer, over 70 years old, screening, survival

## Abstract

**Background:**

Colorectal cancer screening mainly targets a population between 50 and 70 years of age; however, it is inconsistently implemented in people over 70. The aim of this study was to analyze the association between colorectal cancer (CRC) screening, postoperative mortality, and perioperative and oncologic outcomes in a large population of patients over 70 years of age who underwent surgery for CRC.

**Methods:**

Data regarding people over 70 who underwent CRC surgery were retrieved from a nationally validated retrospective database, including four consecutive years (2018–2021) and 81 centers. The patients were divided into two groups according to their participation in the CRC screening program: Screening versus No Screening. The outcomes of the study were 30‐day mortality; urgent, palliative and minimally invasive surgery rates; Clavien‐Dindo ≥ III; advanced oncologic stage; R0 resection and length of hospital stay (LOS). Logistic regression analysis was carried out and adjusted for multiple confounders.

**Results:**

Of the 10,346 patients over 70,676 were in the screening group, and 9670 were in the no screening group. At logistic regression, CRC screening was significantly associated with a reduction in 30‐day mortality (OR 0.41, 95% CI 0.18–0.92, *p* = 0.032), urgent surgery (OR 0.06, 95% CI 0.02–0.14, *p* < 0.001), palliative surgery (OR 0.32, 95% CI 0.19–0.54, *p* < 0.001), Clavien‐Dindo ≥ III complications (OR 0.69, 95% CI 0.51–0.93, *p* = 0.016) and advanced oncologic stage (OR 0.53, 95% CI 0.45–0.62, *p* < 0.001), and a significant increase in R0 resections (OR 3.15, 95% CI 1.67–5.94, *p* < 0.001) and laparoscopic surgery (OR 1.93, 95% CI 1.57–2.38, *p* < 0.001). The crude and adjusted Odds Ratio similarity confirmed this correlation, regardless of the comorbidities and confounders.

**Conclusions:**

Adherence to CRC screening should be further encouraged and standardized for people over 70.

## 1 Introduction

Globally, approximately 1.9 million people are diagnosed with colorectal cancer (CRC) every year, with 900,000 dying as a result of the disease. The majority of these patients are over 70 years of age [1]. This elevated rate of mortality is also due to a lack of CRC screening which mainly targets a population between 50 and 70 years of age [2].

The most recent guidelines recommend extending the CRC screening to the age of 75, while it should be considered individually in people over 75 [3, 4]. However, healthcare regulators implement this age limit inconsistently, often including only people between 50 and 69 in mass CRC screening programs [5, 6, 7]. Extending CRC screening requires large cohort studies which demonstrate a significant reduction in CRC‐related morbidity and mortality in order to justify the harm caused by false‐positive results, their impact on the healthcare system, and their socio‐economic effect. Much doubt exists regarding the lack of benefits of the screening program in terms of reduction in mortality in elderly patients, regardless of the early diagnosis of the disease. Perioperative complications and poor survival account for the major part of the CRC‐related morbidity and mortality, especially in patients over 70 years of age. The correlation between the application of the screening program in these patients and their perioperative outcomes might contribute to clarifying the potential benefits of the screening and strengthening its broader indications.

The aim of this study was to analyze the association between CRC screening, postoperative mortality, and perioperative and oncologic outcomes in a large population of patients over 70 years of age who underwent colorectal surgery for cancer.

## 2 Methods

### 2.1 Study Design and Settings

The COVID‐CRC collaborative study dataset retrospectively included 18,284 consecutive patients who underwent surgery for colorectal cancer in 81 Italian hospitals between January 1st, 2018, and December 31st, 2021 [8, 9, 10, 11]. The study did not impose limits in terms of minimum number of cases, hospital volume and type of center. The data were put into a REDCap (Research Electronic Data Capture; Vanderbilt University) database by a defined team of clinicians in each participating center [12]. A data validator (not involved in the data collection) checked 20% of the cases at the end of the data inclusion period. The Ethics Committees of the coordinating center (08/09/2020; no. 854/2020/Oss/AOUBo) and all of the participating centers approved the COVID‐CRC collaborative study, including subsequent analyses of the dataset. The study was carried out according to the STROBE (Strengthening the reporting of observational studies in epidemiology) guidelines [13].

### 2.2 Participants

**Figure 1:**
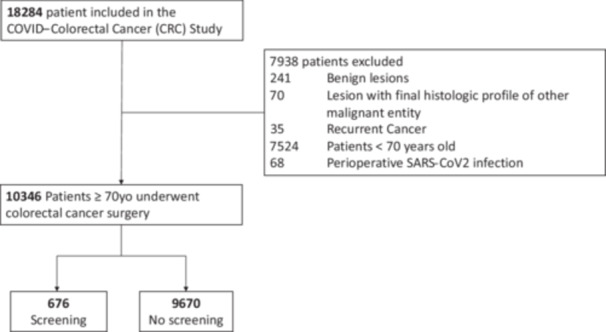
Flowchart of patient selection of the 18,284 patients included in the dataset.

Patients ≥ 70 years of age who underwent surgery in elective, urgent, or emergent settings with curative or palliative intent were included in the study. The exclusion criteria were age under 70 years, benign lesions, final histology profile of other malignant entities, recurrent cancer, and perioperative SARS‐CoV‐2 infection (Flowchart in Figure 1). Potential biases arising from the COVID‐19 pandemic, from different screening pathways of patients with recurrent cancer, from different malignant entities (e.g., neuroendocrine tumors, gastrointestinal stromal tumors, melanomas, lymphomas), and benign tumors were addressed by excluding patients from the analysis. Geographical distribution of the patients according to the region of the hospitals was similar between the two groups. After applying the exclusion criteria (Figure 1), 10,346 patients over 70 years of age were included in the final analysis; they were divided into two groups according to participation in the CRC screening program. The **Screening group** included patients who were asymptomatic and diagnosed with CRC by means of voluntary screening. Colorectal screening consists of a fecal occult blood test (FOBT) and a subsequent colonoscopy in the case of a positive test. The **No Screening group** included patients who did not do the CRC screening, were symptomatic (e.g., occlusion, gastrointestinal bleeding, perforation, weight loss), or had done the FOBT as a means of identifying the origin of anemia. During the study period, the national mass CRC screening was implemented for individuals between 50 and 69, allowing voluntary CRC screening in patient over 70 years old [5, 6, 7].

### 2.3 Variables

The variables collected included the details of the CRC screening, CRC clinical presentation, patient demographics, comorbidities, American Society of Anesthesiologists (ASA) score, use of preoperative chemoradiotherapy, details of the surgery (type, approach, setting, and intraoperative complications), postoperative Intensive Care Unit (ICU) stay, histopathological details, oncologic outcomes, length of hospital stay (LOS), mortality, and complications at 30 days after surgery (according to the Clavien‐Dindo classification) [14].

### 2.4 Study Aims

The primary outcome of the study was to determine whether or not there was a reduction in 30‐day postoperative mortality associated with the screening program. The secondary outcomes were the rates of urgent, palliative, and minimally invasive surgery (laparoscopic or robotic), severe complications (defined as Clavien‐Dindo grade ≥ III), advanced oncologic stage, R0 resection, and LOS. The advanced oncologic stage was defined as a stage greater than IIb (T4 + , N + , M + ), according to the American Joint Committee on Cancer (AJCC) [15]. Urgent surgery was defined as a surgical procedure performed during the index unplanned admission, while palliative surgery was defined as surgery with no curative intent.

### 2.5 Data Analysis

The association between screening status and each selected outcome was initially evaluated using standard univariate analyses (chi‐squared test for the categorical variables; t‐test for the continuous variables). Multivariable analyses were then used to assess whether screening status was an independent predictor of each outcome after adjusting for age, sex, body mass index (BMI), current smoking habit, ASA score, cancer location, comorbidities, and use of multiple drugs. Binomial logistic regression was used for the dichotomous variables, and multiple linear regression was carried out to assess the only continuous variable: LOS. All of the above covariates were included in each model a priori. However, since the variable “current smoking habit” was missing for 2082 patients, and its inclusion or exclusion from the models did not substantially influence screening status estimates, it was excluded from the final models to avoid the loss of power and increase the precision of the estimates.

Each covariate was tested in its original form or transformed if needed (e.g., the presence of comorbidities was dichotomized as “< 2”, or “2 or more”). In addition, each variable included was tested for multicollinearity, potential interaction, and/or quadratic/cubic terms [16].

In the logistic regression analyses, standard diagnostic procedures were adopted to check the validity of the final model: influential observation analysis (Dbeta, change in Pearson chi‐square, and similar), the Hosmer‐Lemeshow test for the goodness of fit, and the C statistic (area under the Receiving Operator Curve). The validity of the final linear regression model was assessed as follows: the assumption of constant error variance was checked graphically, plotting Pearson residuals versus fitted values, and formally, using the Cook‐Weisberg test for heteroskedasticity.

The results of the logistic analyses are presented as odds ratios (OR) and 95% confidence intervals (95% CI), while the results of the linear regression analyses are presented as beta‐coefficients (*β*) and 95% CIs. Statistical significance was defined as a two‐sided *p*‐value < 0.05 for all the analyses carried out using STATA 13.1 (Stata Corp., College Station, TX, U.S.A., 2007).

## 3 Results

The study included 10,346 patients over 70 years of age of which 676 were diagnosed by means of CRC screening, while the other 9670 patients were not.

The characteristics and outcomes of the overall sample and the two groups are shown in Table 1. Patients in the screening group were younger (mean age 76 vs. 79, *p* < 0.001), and had a greater number of males (58% vs. 54%, *p* = 0.072), smokers (12% vs. 9%, *p* = 0.043), and patients with obesity (18% vs. 11%, *p* < 0.001), while they had a lower number of patients having an ASA score > 2 (43% vs. 60%, *p* < 0.001), multiple comorbidities (22% vs. 26%, *p* = 0.038), polypharmacy (8% vs. 10%, *p* = 0.077) and rectal cancer (18% vs. 23%, *p* = 0.008).

**Table 1:** Characteristics and outcomes of patients ≥ 70 years of age overall and by CRC screening.

Variables	Overall sample	Screening	No screening	*p* value^A^
	(*n* = 10,346)	(*n* = 676)	(*n* = 9670)	
Mean age in years (SD)	78.9 (5.7)	76.3 (5.2)	79.1 (5.6)	< 0.001
Male sex, % (*n*)	54.7 (5655)	58.0 (392)	54.4 (5263)	0.072
Current smoking habit, % (*n*)^B^	9.3 (766)	11.7 (63)	9.1 (703)	0.043
Body weight				
Mean BMI (SD)	25.4 (3.8)	26.3 (4.1)	25.3 (3.8)	< 0.001
Obesity (BMI ≥ 30), % (*n*)	11.6 (1195)	17.5 (118)	11.1 (1077)	< 0.001
ASA > 2, % (*n*)	58.4 (6041)	43.1 (291)	59.5 (5750)	< 0.001
Rectal cancer, % (*n*)	23.0 (2375)	18.8 (127)	23.3 (2248)	0.008
≥ 2 comorbidities, % (*n*)	25.2 (2611)	21.9 (148)	25.5 (2463)	0.038
≥ 3 drugs, % (*n*)	10.3 (1064)	8.3 (56)	10.4 (1008)	0.077
30‐day mortality, % (*n*)	3.0 (312)	0.9 (6)	3.2 (306)	0.001
Urgent surgery, % (*n*)	12.5 (1293)	0.7 (5)	13.3 (1288)	< 0.001
Palliative surgery, % (*n*)	7.5 (775)	2.2 (15)	7.9 (760)	< 0.001
Laparoscopic surgery, % (*n*)	69.4 (7182)	83.3 (563)	68.5 (6619)	< 0.001
Stoma formation, % (*n*)^C^	12.4 (1114)	9.8 (60)	12.6 (1054)	0.042
Postoperative complications, % (*n*)				
‐ Overall	32.7 (3380)	27.1 (183)	33.1 (3197)	0.001
‐ Medical	19.8 (2051)	14.0 (95)	20.2 (1956)	< 0.001
‐ Surgical	18.1 (1876)	16.3 (110)	18.3 (1766)	0.19
Clavien‐Dindo grade ≥ III,% (*n*)	10.8 (1118)	6.9 (47)	11.1 (1071)	0.001
Postoperative ICU	19.6 (2025)	13.5 (91)	20.0 (1934)	< 0.001
Advanced stage (IIb + ), % (*n*)	47.3 (4889)	32.5 (220)	48.3 (4669)	< 0.001
AJCC stage 4, % (*n*)^D^	12.5 (1274)	6.6 (44)	12.9 (1230)	< 0.001
≥ 12 lymph nodes, % (*n*)^E^	85.2 (8292)	84.4 (537)	85.3 (7755)	0.55
R0 resection, % (*n*)^F^	94.8 (9707)	98.5 (653)	94.6 (9054)	< 0.001
Mean length of stay in days (SD)	10.4 (9.4)	8.4 (7.2)	10.5 (9.5)	< 0.001

Abbreviations: Advanced stage IIb + : T4 +, N +, M +; AJCC, American Joint Committee on Cancer; ASA, American Society of Anesthesiologists score; BMI, Body mass index; CRC, colorectal cancer; ICU, Intensive care unit; SD, standard deviation.

^A^ T‐test for continuous variables; chi‐squared test for categorical variables.

^B^ Due to missing data, the sample consisted of 8264, 538, and 7726 patients, respectively.

^C^ Due to missing data, the sample consisted of 9012, 615, and 8397patients, respectively.

^D^ Due to missing data, the sample consisted of 10,202, 671, and 9531 patients, respectively.

^E^ Due to missing data, the sample consisted of 9728, 636, and 9092 patients, respectively.

^F^ Due to missing data, the sample consisted of 10,235, 663, and 9572 patients, respectively.

Postoperative 30‐day mortality (primary outcome) was lower in the screening group (0.9% vs. 3.2%, *p* = 0.001). Of the secondary outcomes, the rates of urgent surgery (0.7% vs. 13%, *p* < 0.001), palliative surgery (2% vs. 8%, *p* < 0.001), severe postoperative complications (7% vs. 11%, *p* = 0.001), postoperative need for ICU (14% vs. 20%, *p* < 0.001), advanced oncologic stage (IIb + ) (33% vs. 48%, *p* < 0.001), AJCC stage IV (7% vs. 13%, *p* < 0.001) and LOS (8 days vs. 11 days, *p* < 0.001) were significantly lower in the screening group, while the proportion of R0 resections (99% vs. 95%, *p* < 0.001) and minimally invasive surgery (83% vs. 69%, *p* < 0.001) were significantly higher in the screening group.

At logistic regression, CRC screening was significantly associated with a reduction in 30‐day postoperative mortality (OR 0.41, 95% CI 0.18–0.92, *p* = 0.032), urgent surgery (OR 0.06, 95% CI 0.02–0.14, *p* < 0.001), palliative surgery (OR 0.32, 95% CI 0.19–0.54, *p* < 0.001), Clavien‐Dindo ≥ III postoperative complications (OR 0.69, 95% CI 0.51–0.93, *p* = 0.016) and advanced oncologic stage (OR 0.53, 95% CI 0.45–0.62, *p* < 0.001), and a significant increase of R0 resections (OR 3.15, 95% CI 1.67–5.94, *p* < 0.001) and laparoscopic surgery (OR 1.93, 95% CI 1.57–2.38, *p* < 0.001) (Table 2).

**Table 2:** Multivariable analyses predict the outcomes of patients ≥ 70 years of age who underwent CRC screening versus no screening.

Outcomes	Crude OR (95% CI)	Adj. OR (95% CI)	Adj. *p* A
30‐day mortality	0.27 (0.12–0.62)	0.41 (0.18–0.92)	0.032
30‐day mortality (also adjusted for advanced stage)	0.30 (0.13–0.69)	0.45 (0.20–1.02)	0.056
Urgent surgery	0.05 (0.02–0.12)	0.06 (0.02–0.14)	< 0.001
Palliative surgery	0.27 (0.16–0.45)	0.32 (0.19–0.54)	< 0.001
Laparoscopic surgery	2.30 (1.87–2.82)	1.93 (1.57–2.38)	< 0.001
Postoperative complications			
‐ Overall	0.75 (0.63–0.90)	0.86 (0.72–1.03)	0.11
‐ Medical	0.64 (0.52–0.81)	0.78 (0.62–0.98)	0.033
‐ Surgical	0.87 (0.70–1.07)	0.91 (0.73–1.13)	0.4
Clavien‐Dindo grade ≥III	0.60 (0.44–0.81)	0.69 (0.51–0.93)	0.016
Postoperative ICU	0.62 (0.50–0.78)	0.77 (0.61–0.98)	0.033
Advanced stage (IIb + )	0.52 (0.44–0.61)	0.53 (0.45–0.62)	< 0.001
AJCC stage 4, % (*n*)^C^	0.47 (0.35–0.65)	0.48 (0.35–0.65)	< 0.001
R0 resection^E^	3.74 (1.99–7.02)	3.15 (1.67–5.94)	< 0.001

	Crude Reg. Coeff. (95% CI)	Adj. Reg. Coeff. (95% CI)	Adj. *p* A
Length of stay in days	2.1 (−2.8; −1.3)	1.6 (−2.3; −0.9)	< 0.001

Abbreviations: Adj, Adjusted; Advanced stage (IIb +): T4 +, N +, M +; AJCC, American Joint Committee on Cancer; CI, Confidence Interval; Coeff., Coefficient; CRC, colorectal cancer; ICU, Intensive care unit; OR, Odds ratio; Reg., Regression.

^A^ Logistic model for categorical variables; multiple regression models for continuous variables. All the models have been adjusted for age, gender, BMI, ASA group, cancer location (rectum or not), multiple comorbidities, and drugs.

^C^ Due to missing data, the model included only 10,202 observations.

^E^ Due to missing data, the model included only 10,235 observations.

Outcomes and multivariate analysis of patients over 75 years of age were reported in the supporting material (Tables S1 and S2) as this threshold is adopted in other countries.

The causes of 30‐day mortality were analyzed. In the screening group (*n* = 6) they were a composite of sepsis and multi‐organ failure following anastomotic leak (3/6, 50%), pneumonia (2/6, 33%), and bleeding (1/6, 17%). In the no screening group, the 30‐day mortality (*n* = 306) was affected more by medical (211/306, 69%) than surgical (95/306, 31%) complications. Among them, the most common medical complications were respiratory failure (93/306, 30%), sepsis (64/306, 21%), anemia (56/306, 18%), acute kidney injury (48/306, 16%), pneumonia (47/306, 15%), myocardial infarction (26/306, 9%), deep venous thrombosis and pulmonary embolism (15/306, 5%), and stroke (6/306, 2%). The most common surgical complications leading to 30‐day mortality were anastomotic leak (59/306, 19%), abscesses/peritonitis (19/306, 6%), postoperative ileus (4/306, 1%), and intestinal occlusion (3/306, 1%).

## 4 Discussion

The present study showed that the CRC screening program was associated with reduced mortality, morbidity, urgent and palliative surgery, advanced oncologic stage, and increased R0 resections and laparoscopic surgery, specifically in patients over 70 years of age. The crude and adjusted Odds Ratio similarity confirmed this correlation, regardless of the comorbidities and potential confounders. These findings support the implementation of CRC screening programs in the elderly population. Regardless of the long‐term survival rate after surgery, which was not assessed in the present study and could be affected by several confounders, such a significant improvement in all postoperative outcomes should be considered to be solid proof for the strong association between screening and the overall survival of patients of 70 years of age and over affected by colorectal cancer.

Previous studies and recent guidelines have recommended CRC screening through age 75, and in people over 75 without significant comorbidities [3, 4, 17, 18, 19]. However, national and regional healthcare systems implement this age limit differently. In fact, this study was carried out on a national healthcare system in which mass CRC screening is offered to people between 50 and 69 [5, 6, 7].

In this large cohort study, over‐70‐year‐old CRC screening with FOBT showed improved postoperative survival and oncologic outcomes. Extending CRC screening requires studies that balance all the potential effects (reduction of CRC incidence and mortality, the harm of false‐positive results, adverse events in screening, healthcare readiness, and socio‐economic evaluation). According to the data presented, one should expect a correlation between the decreased risks of complications, urgent surgery, postoperative ICU admission, and LOS with decreased stress on the healthcare system and reduced costs [18, 19]. Since the rate of patients over 70 years of age was approximately 60% of all the 18,284 patients included in the COVID‐CRC dataset (Figure 1), the implementation of CRC screening is even more relevant in order to extend its benefits to the population affected by the highest incidence of CRC [1].

The present study has some limitations. First, the retrospective and observational nature, and the voluntary participation of the centers may have introduced selection bias, potentially making the study population unrepresentative of the general population of patients undergoing surgery for CRC. However, this effect was mitigated by the large sample size which included centers having varying volumes, and the accurate patient inclusion carried out to reduce the effect of confounders, such as perioperative SARS‐CoV‐2 infection and different screening pathways.

Second, patients who underwent non‐operative management and/or endoscopic treatment, and patients having unresectable metastatic cancer were not included in the dataset. This could attenuate the negative effect of non‐performing CRC screening on mortality and on palliative surgery.

Third, this collaborative study dataset analyzed the 30‐day postoperative outcomes, preventing the exploration of long‐term outcomes. However, reduced 30‐day mortality, AJCC stage, and R0 resection rates in patients over 70 years of age undergoing CRC screening represented valid surrogates impacting the tendency of the long‐term outcomes.

Fourth, results were not adjusted for socioeconomic status. However, its effect should be little considering the cost of a voluntary CRC screening in a universal healthcare system that provides non‐essential medical services (like FOBT after 70 years old) through income‐based healthcare co‐payments and free care in case of oncologic diagnosis. Also, cost‐effectiveness was not reported in the present study, which will be a topic for further general population studies.

In conclusion, considering the findings of the present study, adherence to CRC screening programs should be further encouraged and standardized in people over 70 years of age.

## List of COVID‐CRC Clinical Investigators

Angela Romano, Angela Belvedere, Antonio Lanci Lanci, Daniele Parlanti, Gabriele Vago, Anna Paola Pezzuto, Anna Canavese, Gerti Dajti, Stefano Cardelli, Caterina Catalioto, Iris S. Russo, Tommaso Violante, Daniele Morezzi, Ludovica Maurino, Eleonora Filippone, Dajana Cuicchi **(** ***Surgery of the Alimentary Tract, IRCCS Azienda Ospedaliero‐Universitaria di Bologna, Bologna, Italy) (Department of Medical and Surgical Sciences, Alma Mater Studiorum University of Bologna, Bologna, Italy***); Elio Jovine, Raffaele Lombardi, Chiara Cipressi, Maria F. Offi, Cristina Larotonda, Silvana B. Puglisi (***Chirurgia A e d'Urgenza IRCCS AOU c/o OM, IRCCS Azienda Ospedaliero‐Universitaria di Bologna, Bologna, Italy) (Department of Medical and Surgical Sciences, Alma Mater Studiorum University of Bologna, Bologna, Italy***); Augusto Barbosa, Roberto Vaiana, Paolo M. Bianchi, Carlo Tonti, Claudio Codignola (***Fondazione Poliambulanza Brescia***); Luigi Zorcolo, Angelo Restivo, Simona Deidda, Marcello E. Marchetti, Luca Ippolito (***Unità operativa di Chirurgia Coloproctologica ‐ AOU Cagliari***); Gaya Spolverato, Salvatore Pucciarelli, Francesco Marchegiani, Giacomo Ghio, Gaia Zagolin, Dajana Glavas, Monica Tomassi ***(Department of Surgical Oncological and Gastroenterological Sciences, University of Padova ‐ General Surgery 3, Azienda Ospedale Università di Padova***); Ugo Elmore, Lorenzo Gozzini, Riccardo Calef, Francesco Puccetti, Andrea Cossu, Andrea Vignali (***Gastrointestinal Surgery Division, IRCCS San Raffaele Hospital, Milan***); Marco E. Allaix, Gaspare Cannata, Erica Lombardi, Carlo A. Ammirati, Chiara Piceni (***AOU Città della Salute e della Scienza, Turin, Italy***), Piero Buccianti, Riccardo Balestri, Marco Puccini, Daniele Pezzati, Roberto d'Ischia, Vito F. Asta, Benedetta Sargenti, Giacomo Taddei, Federica Bonari, Giulia Boni (***Azienda Ospedaliero‐Universitaria Pisana***); Alessandro Ferrero, Michela Mineccia, Federica Gonella, Marco Palisi, Francesco Danese, Valeria Cherubini, Serena Perotti (***Azienda Sanitaria Ospedaliera Ordine Mauriziano Umberto I°, Torino***); Michele Carvello, Fabio Carbone, Antonio Luberto, Eleonora Calafiore, Francesca De Lucia, Matteo Sacchi ***(IRCCS Humanitas Research Hospital, Rozzano, Milan, Italy)***; Diego Sasia, Maria C. Giuffrida, Edoardo Ballauri, Mathieu Cardile, Serena Armentano, Elsa Beltrami, Gabriele Preve, Barbara Vercellone (***Santa Croce and Carle Hospital, Cuneo***); Marta Mozzon, Cristina Folliero, Chiara Lirusso, Massimo Vecchiato, Antonio Ziccarelli, Davide Gattesco, Luisa Moretti, Sara Crestale (***Chirurgia Generale, Azienda ospedaliero‐universitaria S. Maria della Misericordia Udine‐ASUFC***); Filippo Banchini, Patrizio Capelli, Andrea Romboli, Gerardo Palmieri, Luigi Conti, Nicholas Rizzi (***UO Chirurgia Generale Vascolare di Piacenza***); Deborah Bonfili (***Dipartimento di Chirurgia, Università degli Studi di Parma***); Paola Germani, Edoardo Osenda, Sara Cortinovis, Carlotta Giunta, Stefano Fracon, Hussein Abdallah, Selene Bogoni (***General surgery department, University Hospital of Trieste***); Nazario Portolani, Riccardo Nascimbeni, Sarah Molfino, Guido A. M. Tiberio, Ilenia Garosio, Giulia Lamperti, Diego Rigosa (***U.O. Chirurgia Generale 3 ‐ ASST Spedali Civili Brescia, Università di Brescia***); Giorgio Ercolani, Leonardo Solaini, Davide Cavaliere, Andrea Avanzolini, Fabrizio D'Acapito, Leonardo L. Chiarella, Daniela Di Pietrantonio, Domenico Annunziata (***Chirurgia generale e TOA, Ospedale Morgagni‐Pierantoni, Forlì***); Roberta Piccolo, Mario Sorrentino, Mauro Pansini, Alessandro Cojutti, Michele Graziano, Francesco Callegari (***U.O. Chirurgia Generale Ospedale di Latisana‐Palmanova, Azienda Ospedaliera Universitaria Friuli Centrale***); Laura Balzarotti, Vitale R. Dameno, Antonio Cattaneo, Giuliano Santolamazza, Caterina Altieri, Riccardo Magarini (***Ospedale civile “G. Fornaroli”, Magenta***); Tommaso Dominioni, Luigi Pugliese, Andrea Peri, Marta Botti, Benedetta Sargenti, Francesco Salvetti (***Department of Surgery, University of Pavia and Fondazione IRCCS Policlinico San Matteo***); Elisa Cassinotti, Ludovica Baldari, Valentina Messina, Vera D'Abrosca (***SC Chirurgia Generale e Mini‐invasiva, Fondazione IRCCS Ca' Granda Ospedale Maggiore Policlinico ‐ Milano***); Pasquale Cianci, Rocco Tumolo, Domenico Gattulli, Enrico Restini, Marina Minafra, Maria Grazia Sederino, Bernardino Bottalico (***UOC Chirurgia Generale, Ospedale Lorenzo Bonomo, Andria***); Pierluigi Pilati, Boris Franzato, Genny Mattara, Ottavia De Simoni, Andrea Barina, Marco Tonello (***Unit of Surgical Oncology of Digestive Tract, Veneto Institute of Oncology IOV‐IRCCS, Padova, Italy***); Andrea Muratore, Marcello Calabrò, Nicoletta S. Federico Pipitone, Bruno Cuzzola, Elena Herranz Van Nood, Mariangela Azzellino (***Chirurgia Generale Ospedale E. Agnelli, Pinerolo***); Nicola Passuello, Alvise Frasson, Enzo Mammano, Luca Faccio, Fabrizio Vittadello, Alice Bressan, Giacomo Sarzo (***U.O.C. Chirurgia Generale OSA, io DIDAS Chirurgia, Azienda Ospedale‐Università Padova***); Nicolò Tamini, Massimo Oldani, Luca Cigagna, Francesca Carissimi, Giulia De Carlo, Edoardo Baccalini, Luca Nespoli (***UO Chirurgia 1, Fondazione IRCCS San Gerardo dei Tintori, Università di Milano‐Bicocca***); Alessio Giordano, Stefano Cantafio, Lucrezia Grifoni, Davide Matani, Serena Livi (***UO di Chirurgia Generale, Nuovo Ospedale “S. Stefano”, Azienda ASL Toscana Centro***); Daniele Delogu, Fabrizio Scognamillo, Antonio Marrosu, Luca Guerrini (***Patologia Chirurgica AOU, Sassari***); Giampaolo Ugolini, Federico Ghignone, Giacomo Frascaroli, Nicola Albertini, Davide Zattoni, Giovanni Taffurelli, Isacco Montroni (***UO Chirurgia Generale di Ravenna‐Faenza, AUSL Romagna***); Francesco Colombo, Piergiorgio Danelli, Andrea Bondurri, Anna Maffioli, Alessandro Bonomi, Isabella Pezzoli, Francesco Cammarata (***Division of General Surgery ‐ L. Sacco University Hospital‐ Milano***); Orlando Goletti, Mattia Molteni, Alberto Assisi, Giorgio Quartierini (***Chirurgia Generale Humanitas Gavazzeni Bergamo, Italy***); Corrado Da Lio, Daunia Verdi, Isabella Mondi, Claudia Peluso, Lorenzo Macchi, (***Department of General Surgery, Mirano Hospital, Venice***); Marta Tanzanu, Federico Zanzi, Sara Pellegrini (***Chirurgia d'Urgenza, Santa Maria delle Croci ‐ Ravenna***); Jacopo Andreuccetti, Rossella D'Alessio, Giusto Pignata, Michele De Capua, Ilaria Canfora, Luca Ottaviani (***General Surgery 2, ASST Spedali Civili of Brescia***); Pasquale Lepiane, Andrea Balla, Antonio De Carlo, Federica Saraceno, Rosa Scaramuzzo, Anna Guida, Daniele Aguzzi (***Ospedale San Paolo, Civitavecchia, Roma***); Paolo Bellora, Sergio Gentilli, Manuela Monni, Herald Nikaj (***Clinica Chirurgica Ospedale Maggiore della Carità ‐ Novara***); Nicola Cillara, Alessandro Cannavera, Antonello Deserra, Carla Margiani, Roberta Cabula (***UOC Chirurgia Generale PO Santissima Trinità ASL Cagliari***); Manuela Dettori ***(Oncologia Medica, PO Businco, ARNAS Cagliari)***; Giulia Gramignano ***(SSD Oncologia, PO Nostra Signora di Bonaria San Gavino, ASL Medio Campidano)**;* Giovanni Lezoche, Monica Ortenzi, Elena S. Orlandoni, Federica Curzi, Francesca Vitali, Perla Capomagi, Miriam Palmieri (***Clinica di Chirurgia Generale e d'urgenza, Ancona Torrette***); Mario Giuffrida, Paolo Del Rio, Elena Bonati, Tommaso Loderer, Federico Cozzani, Matteo Rossini, Stefano Agnesi, (***Clinica Chirurgica Generale ‐ AOU Parma***); Gabriella T. Capolupo, Marco Caricato, Filippo Carannante, Gianluca Mascianà, Martina Marrelli, Valentina Miacci, Sara Lauricella (***UOC Chirurgia colorettale, Fondazione Policlinico Campus Bio Medico, Roma***); Valeria Tonini, Maurizio Cervellera, Salvatore Pisconti, Concetta Lozito, Juliana Shahu, Claudia Mongelli, Giulia Morelli, Lodovico Sartarelli (***Ospedale Santissima Annunziata, Taranto***); Giuseppe S. Sica, Leandro Siragusa, Giulia Bagaglini, Andrea M. Guida, Marzia Franceschilli, Vittoria Bellato, Cristina Fiorani (***Policlinico Tor Vergata, Roma***); Antonio Taddei, Matteo Risaliti, Ilenia Bartolini, Maria N. Ringressi, Luca Tirloni (***Azienda Ospedaliero Universitaria Careggi, Firenze***); Letizia Laface, Emmanuele Abate, Massimiliano Casati, Pietro Gobbi (***Ospedale Vittorio Emanuele III Carate Brianza***); Enrico Opocher, Nicolò M. Mariani, Andrea Pisani Ceretti, Marco Giovenzana, Beatrice Giuliani, Martina Sironi (***ASST Santi Paolo e Carlo, Milano***); Ugo Grossi, Giacomo Zanus, Giulio Aniello Santoro, Marco Brizzolari, Eugenio De Leo, Simone Novello, Krizia Aquilino, Francesco Milardi (***II Surgery Unit, Regional Hospital Treviso, DISCOG, University of Padua, Italy***); Stefano Olmi, Matteo Uccelli, Marta Bonaldi, Giovanni C. Cesana, Marco Bindi (***Policlinico San Marco GSD, Zingonia***); Raffaele Galleano, Antonio Langone, Massimiliano Botto, Angelo Franceschi, Elena Gambino (***Ospedale San Paolo Savona***); Maurizio Ronconi, Silvia Casiraghi, Giovanni Casole, Salvatore L. Ciulla (***Ospedale di Gardone V.T. ‐ ASST Spedali Civili di Brescia***); Giovanni Terrosu, Sergio Calandra, Edoardo Scarpa, Vittorio Cherchi, Giacomo Calini, Lisa Martinuzzo, Lucrezia Clocchiatti, Davide Muschitiello (***Clinica Chirurgica, Azienda Sanitaria Universitaria Friuli Centrale ASUFC, Udine***); Andrea Romanzi, Barbara Vignati, Alberto Vannelli, Roberta Scolaro, Maria Milanesi, Fabrizio Rossi (***Department of General Surgery, Valduce Hospital, Como, Italy***); Giuseppe Canonico, Alessandro Anastasi, Tommaso Nelli, Marco Barlettai, Riccardo Fratarcangeli, Carmela Di Martino, Andrea Damigella, Elvira Adinolfi (***Ospedale San Giovanni di Dio, Firenze***); Arianna Birindelli, Lucio Taglietti, Sara E. Dester (***UOC Chirurgia ‐ Ospedale di Esine (BS) ‐ ASST Valcamonica ‐ Italy***); Francesco Fleres, Eugenio Cucinotta, Francesca Viscosi, Santino A. Biondo, Giorgio Badessi, Nivia Catarsini, Carmelo Mazzeo (***AOU G Martino Policlinico di Messina, Department of General and Emergency Surgery ‐ Italy***); Daniela Rega, Paolo Delrio, Carmela Cervone, Alessia Aversano, Silvia De Franciscis, Massimiliano Di Marzo, Bruno Marra, Ugo Pace (***Colorectal Surgical Oncology, Department of Abdominal Oncology, Istituto Nazionale Tumori‐IRCCS “Fondazione G. Pascale”, Naples, Italy***); Antonio Amato, Paola Batistotti, Elisa Mina, Alberto Serventi (***SC Chirurgia Generale Imperia***); Pierfrancesco Lapolla, Andrea Mingoli, Paolo Sapienza, Gioia Brachini, Bruno Cirillo, Enrico Fiori, Daniele Crocetti, Ilaria Clementi (***Policlinico Umberto I Sapienza Università di Roma***); Gennaro Martines, Arcangelo Picciariello, Giovanni Tomasicchio, Rigers Dibra, Giuseppe Trigiante, Marcella Rinaldi, Giuliano Lantone (***Chirurgia Generale “M. Rubino” Azienda Ospedaliero Universitaria Policlinico Bari Italy***); Alberto Porcu, Teresa Perra, Antonio M. Scanu, Claudio F. Feo, Alessandro Fancellu, Maria L. Cossu, Giorgio C. Ginesu, (***Azienda Ospedaliero Universitaria di Sassari, Italia***); Alberto Patriti, Diego Coletta, Filippo Petrelli, Paola A. Greco, Claudia Spadoni, Giovanna Cassiani, Federica Bianchini (***AO Ospedali Riuniti Marche Nord***); Marco Arganini, Matteo Bianchini, Bruno Perotti, Matteo Palmeri, (***Ospedale Unico della Versilia ‐ Azienda Usl Toscana Nord‐ovest***); Stefano Scabini, Selene Deiana, Giacomo Carganico, Davide Pertile, Domenico Soriero, Emanuela Fioravanti, Beatrice Sperotto (***Unità Operativa Chirurgia Generale ad Indirizzo Oncologico ‐ IRCCS Ospedale Policlinico San Martino, Genova***); Bruno Nardo, Daniele Paglione, Veronica Crocco, Marco Doni, Mariasara Osso, Roberto Perri (***U.O.C. di Chirurgia Generale “Falcone” ‐ Azienda Ospedaliera di Cosenza ‐ Università della Calabria***); Gianluca M. Sampietro, Carlo Corbellini, Leonardo Lorusso, Carlo A. Manzo, Maria Cigognini, Caterina Baldi (***Division of Surgery, Rho Memorial Hospital ‐ ASST Rhodense ‐ Rho, Milan***); Giuseppe Palomba, Giovanni Aprea, Marianna Capuano, Raffaele Basile (***AOU Federico II di Napoli ‐ UOC chirurgia endoscopica***); Roberta Tutino, Marco Massani, Laura Marinelli, Nicola Canitano (***Chirurgia 1 ‐ Azienda ULSS2 Marca Trevigiana ‐ Ospedale Regionale di Treviso***); Tiziana Pilia, Mauro Podda, Adolfo Pisanu, Valentina Murzi, Silvia Incani, Federica Frongia, Giuseppe Esposito (***Policlinico di Monserrato, Chirurgia d'urgenza, Cagliari***); Gaetano Luglio, Francesca P. Tropeano, Gianluca Pagano, Eduardo Spina, Giuseppe De Simone, Michele Cricrì (***Azienda Ospedaliera Universitaria Federico II***); Fausto Catena, Carlo Vallicelli, Nicola Zanini, Diana Ronconi, Francesco Favi, Carlo Mazzucchelli, Girolamo Convertini (***Chirurgia Generale e d'Urgenza, Ospedale Bufalini di Cesena, AUSL della Romagna***); Leonardo Vincenti, Valeria Andriola, Cinzia Bizzoca (***Chirurgia Generale Ospedaliera, Policlinico di Bari***); Carlo V. Feo, Nicolò Fabbri, Marta Fazzin, Antonio Pesce, Silvia Gennari, Marco Torchiaro, Silvia Severi (***Azienda Unità Sanitaria Locale di Ferrara, Università di Ferrara***); Alice Frontali, Greta Bracchetti, Stefano Granieri, Christian Cotsoglou (***General Surgery Unit, ASST Vimercate, Vimercate, Italy***); Massimo Carlini, Giorgio Lisi, Domenico Spoletini, Maria R. Mastrangeli, Michela Campanelli (***UOC Chirurgia Generale, Ospedale Sant'Eugenio, Roma, Italia***); Michele Manigrasso, Marco Milone, Giovanni D. De Palma, Sara Vertaldi, Alessia Chini, Francesco Maione, Alessandra Marello (***Department of Clinical Medicine and Surgery, “Federico II” University of Naples, Naples, Italy***); Francesco Selvaggi, Guido Sciaudone, Lucio Selvaggi, Francesco Menegon Tasselli, Giacomo Fuschillo, Lidia Oddis (***Università della Campania Luigi Vanvitelli, Napoli***); Simona Grande, Michele Grande (***UOSD Chirurgia d'urgenza Tor Vergata***); Simona Ascanelli, Laura Chimisso, Filippo Aisoni, Eleonora Rossin, Francesco Pepe, Francesco Marchetti (***UO Chirurgia 2 Azienda Ospedaliero‐Universitaria Ferrara***); Biagio Picardi, Stefano Rossi, Simone Rossi Del Monte, Matteo Picarelli, Irnerio A. Muttillo (**Chirurgia Generale e d'Urgenza Ospedale** ***San Filippo Neri ASL Roma 1***); Carlo Ratto, Angelo A. Marra, Angelo Parello, Francesco Litta, Paola Campennì, Veronica De Simone (***Fondazione Policlinico Universitario Agostino Gemelli, IRCCS, Roma***); Francesco Pata ***(Department of Surgery, Nicola Giannettasio Hospital, Corigliano‐Rossano, Italy) (La Sapienza University, Rome, Italy);*** Cristiana Riboni ***(EOC Ospedale Regionale di Lugano, Lugano, Switzerland);*** Emanuele Rausa ***(Unit of Hereditary Digestive Tumors, Fondazione IRCCS‐National Cancer Institute, Milan, Italy)*** Valerio Celentano ***(Chelsea and Westminster Hospital NHS Foundation Trust, London, United Kingdom) (Department of Surgery and Cancer, Imperial College, London, United Kingdom)***.

## Funding

The authors received no specific funding for this work.

## Conflicts of Interest

The authors declare no conflicts of interest.

## Synopsis

This large national cohort study showed that the CRC screening program in patients over 70 years of age was associated with reduced mortality, morbidity, urgent and palliative surgery, advanced oncologic stage, and increased R0 resections and laparoscopic surgery. Adherence to CRC screening programs should be further encouraged and standardized for people over 70.

## Supporting information


**Table S1:** Subgroup analysis of the outcomes of patients ≥ 75 years of age, overall and by screening participation. **Table S2:** Subgroup multivariable analyses predicting the outcomes of patients ≥75 years who underwent CRC screening versus no screening (sample restricted to the 7607 subjects aged ≥75 years).

## Data Availability

The data will be made available upon reasonable request to the corresponding author.

## References

[1] Cancer Today, accessed February 5, 2025, https://gco.iarc.who.int/today/.

[2] D. Gornick , A. Kadakuntla , A. Trovato , R. Stetzer , and M. Tadros , “Practical Considerations for Colorectal Cancer Screening in Older Adults,” World Journal of Gastrointestinal Oncology 14, no. 6 (2022): 1086–1102, 10.4251/wjgo.v14.i6.1086. 35949211 PMC9244986

[3] A. M. D. Wolf , E. T. H. Fontham , T. R. Church , et al., “Colorectal Cancer Screening for Average‐Risk Adults: 2018 Guideline Update From the American Cancer Society,” CA: A Cancer Journal for Clinicians 68, no. 4 (2018): 250–281, 10.3322/caac.21457. 29846947

[4] A. Shaukat , C. J. Kahi , C. A. Burke , L. Rabeneck , B. G. Sauer , and D. K. Rex , “ACG Clinical Guidelines: Colorectal Cancer Screening 2021,” American Journal of Gastroenterology 116, no. 3 (2021): 458–479, 10.14309/ajg.0000000000001122. 33657038

[5] Salute M. della . Screening per il cancro del colon retto, accessed February 6, 2025, https://www.salute.gov.it/portale/tumori/dettaglioContenutiTumori.jsp?id=5541&area=tumori&menu=screening.

[6] EpiCentro . Screening colorettale dati sorveglianza Passi, accessed February 6, 2025, https://www.epicentro.iss.it/passi/dati/screeningcolorettale.

[7] Screening tumore colon retto, l'Emilia‐Romagna amplia l'offerta, da gennaio il test sarà gratuito dai 50 ai 74 anni di età. Salute, accessed February 6, 2025, https://salute.regione.emilia-romagna.it/notizie/regione/2025/gennaio/screening-tumore-colon-retto-l2019emilia-romagna-amplia-l2019offerta-da-gennaio-il-test-sara-gratuito-dai-50-ai-74-anni-di-eta.

[8] M. Rottoli , A. Gori , G. Pellino , et al., “Colorectal Cancer Stage at Diagnosis Before versus During the COVID‐19 Pandemic in Italy,” JAMA Network Open 5, no. 11 (2022): e2243119, 10.1001/jamanetworkopen.2022.43119. 36409496 PMC9679872

[9] M. Rottoli , A. Spinelli , G. Pellino , et al., “Effect of Centre Volume on Pathological Outcomes and Postoperative Complications After Surgery for Colorectal Cancer: Results of A Multicentre National Study,” British Journal of Surgery 111, no. 1 (2024): znad373, 10.1093/bjs/znad373. 37963162 PMC10771132

[10] M. Rottoli , G. Pellino , A. Spinelli , et al., “Impact of COVID‐19 on the Oncological Outcomes of Colorectal Cancer Surgery in Northern Italy in 2019 and 2020: Multicentre Comparative Cohort Study,” BJS Open 6, no. 1 (2022): zrab139, 10.1093/bjsopen/zrab139. 35143629 PMC8830755

[11] M. Rottoli , A. Gori , G. Pellino , M. E. Flacco , A. Spinelli , and G. Poggioli , “Is The Significant Risk of Perioperative Complications Associated With Radical Surgery Following Non‐Curative Endoscopic Submucosal Dissection For Early Colorectal Cancer Still Acceptable?,” Gut 73, no. 2 (2024): 385–388, 10.1136/gutjnl-2022-328076. 36697206

[12] P. A. Harris , R. Taylor , R. Thielke , J. Payne , N. Gonzalez , and J. G. Conde , “Research Electronic Data Capture (REDCap)‐‐A Metadata‐Driven Methodology and Workflow Process for Providing Translational Research Informatics Support,” Journal of Biomedical Informatics 42, no. 2 (2009): 377–381, 10.1016/j.jbi.2008.08.010. 18929686 PMC2700030

[13] E. Von Elm , D. G. Altman , M. Egger , S. J. Pocock , P. C. Gøtzsche , and J. P. Vandenbroucke , “Strengthening the Reporting of Observational Studies in Epidemiology (STROBE) Statement: Guidelines for Reporting Observational Studies,” BMJ (London) 335, no. 7624 (2007): 806–808, 10.1136/bmj.39335.541782.AD. PMC2034723 17947786

[14] P. A. Clavien , J. Barkun , M. L. de Oliveira , et al., “The Clavien‐Dindo Classification of Surgical Complications: Five‐Year Experience,” Annals of Surgery 250, no. 2 (2009): 187–196, 10.1097/SLA.0b013e3181b13ca2. 19638912

[15] M. Amin , S. Edge , F. Greene , et al., AJCC Cancer Staging Manual 8th Edition (2017).

[16] The Role of Needle Fear in Pediatric Flu Vaccine Hesitancy: A Cross‐Sectional Study in Bologna Metropolitan Area – PubMed, accessed February 7, 2025, https://pubmed.ncbi.nlm.nih.gov/36146466/. 10.3390/vaccines10091388 PMC9506010 36146466

[17] W. Ma , K. Wang , L. H. Nguyen , et al., “Association of Screening Lower Endoscopy With Colorectal Cancer Incidence and Mortality in Adults Older Than 75 Years,” JAMA Oncology 7, no. 7 (2021): 985–992, 10.1001/jamaoncol.2021.1364. 34014275 PMC8138747

[18] J. C. Glasbey , T. E. Abbott , A. Ademuyiwa , et al., “Elective Surgery System Strengthening: Development, Measurement, and Validation of the Surgical Preparedness Index Across 1632 Hospitals in 119 Countries,” Lancet 400, no. 10363 (2022): 1607–1617, 10.1016/S0140-6736(22)01846-3. 36328042 PMC9621702

[19] M. C. Ramos , J. Passone , A. Lopes , A. V. Safatle‐Ribeiro , U. Ribeiro Júnior , and P. C. de Soárez , “Economic Evaluations of Colorectal Cancer Screening: A Systematic Review and Quality Assessment,” Clinics (Sao Paulo, Brazil) 78 (2023): 100203, 10.1016/j.clinsp.2023.100203. 37099816 PMC10182269

