# Supplementary material for: Association Between Colorectal Cancer Screening and Survival in Patients Older Than 70 Years: Results of A National Multicenter Retrospective Study
Source: J Surg Oncol. 2026 Feb 22;133(5):572–9. doi: 10.1002/jso.70206 (PMC13058403; doi:10.1002/jso.70206)
Supplement: Supplementary file 1 — Table S1: Subgroup analysis of the outcomes of patients ≥ 75 years of age, overall and by screening participation. Table S2: Subgroup multivariable analyses predicting the outcomes of patients ≥75 years who underwent CRC screening versus no screening (sample restricted to the 7607 subjects aged ≥75 years). [file JSO-133-572-s001.doc]

**Supplementary tables**

**Table S1**. Subgroup analysis of the outcomes of patients ≥ 75 years of age, overall and by screening participation.

| **Variables** | **Overall sample** | **Screening** | **No screening** | **p-value ^A^** |
| --- | --- | --- | --- | --- |
|  | (n=7607) | (n=358) | (n=7249) |  |
|  |  |  |  |  |
| 30-day mortality, % (n) | 3.7 (282) | 1.4 (5) | 3.8 (277) | 0.018 |
|  |  |  |  |  |
| Urgent surgery, % (n) | 13.8 (1051) | 0.3 (1) | 14.5 (1050) | <0.001 |
|  |  |  |  |  |
| Palliative surgery, % (n) | 8.0 (609) | 2.8 (10) | 8.3 (599) | <0.001 |
|  |  |  |  |  |
| Laparoscopic surgery, % (n) | 67.7 (5150) | 81.3 (291) | 67.0 (4859) | <0.001 |
|  |  |  |  |  |
| Stoma formation, % (n) ^B^ | 11.2 (742) | 8.0 (26) | 11.4 (716) | 0.061 |
|  |  |  |  |  |
| Postoperative complications, % (n) |  |  |  |  |
| - Overall | 34.5 (2624) | 31.8 (114) | 34.6 (2510) | 0.28 |
| - Medical | 21.7 (1651) | 16.2 (58) | 22.0 (1593) | 0.010 |
| - Surgical | 18.4 (1396) | 19.3 (69) | 18.3 (1327) | 0.64 |
|  |  |  |  |  |
| Clavien-Dindo grade ≥III, % (n) | 11.3 (859) | 9.2 (33) | 11.4 (826) | 0.204 |
|  |  |  |  |  |
| Postoperative ICU | 21.2 (1616) | 16.5 (59) | 21.5 (1557) | 0.024 |
|  |  |  |  |  |
| Advanced stage (IIb+), % (n) | 47.1 (3585) | 33.8 (121) | 47.8 (3464) | <0.001 |
|  |  |  |  |  |
| AJCC stage 4, % (n) ^C^ | 12.1 (912) | 8.1 (29) | 12.3 (883) | 0.019 |
|  |  |  |  |  |
| ≥12 lymph nodes, % (n) ^D^ | 85.5 (6118) | 85.9 (286) | 85.5 (5832) | 0.84 |
|  |  |  |  |  |
| R0 resection, % (n) ^E^ | 94.7 (7131) | 97.7 (344) | 94.6 (6787) | 0.010 |
|  |  |  |  |  |
| Mean length of stay, days (SD) | 10.6 (9.1) | 8.9 (7.0) | 10.7 (9.1) | <0.001 |
|  |  |  |  |  |

ICU: Intensive care unit. Advanced stage IIb+: T4+, N+, M+; AJCC: American Joint Committee on Cancer; SD: standard deviation.

^A^ T-test for continuous variables; chi-squared test for categorical variables.

^B^ Because of missing data, the overall sample consisted of 9012, 615 and 8397 individuals, respectively.

^C^ Because of missing data, the overall sample consisted of 10,202, 671 and 9531 individuals, respectively.

^D^ Because of missing data, the overall sample consisted of 9728, 636 and 9092 individuals, respectively.

^E^ Because of missing data, the overall sample consisted of 10,235, 663 and 9572 individuals, respectively.

**Table S2**. Subgroup multivariable analyses predicting the outcomes of patients ≥75 years who underwent CRC screening versus no screening (sample restricted to the 7607 subjects aged ≥75 years).

| **Outcomes** | **Crude OR**  **(95% CI)** | **Adj. OR**  **(95% CI)** | **Adj. p ^A^** |
| --- | --- | --- | --- |
|  |  |  |  |
| 30-day mortality | 0.36 (0.15-0.87) | 0.44 (0.18-1.07) | 0.071 |
| 30-day mortality (also adjusted for Advanced stage) | -- | 0.48 (0.19-1.17) | 0.107 |
|  |  |  |  |
| Urgent surgery | 0.02 (0.00-0.12) | 0.02 (0.00-0.13) | <0.001 |
|  |  |  |  |
| Palliative surgery | 0.32 (0.17-0.60) | 0.37 (0.19-0.69) | 0.002 |
|  |  |  |  |
| Laparoscopic surgery | 2.14 (1.63-2.80) | 1.91 (1.45-2.51) | <0.001 |
|  |  |  |  |
| Postoperative complications |  |  |  |
| - Overall | 0.88 (0.70-1.11) | 0.95 (0.75-1.19) | 0.651 |
| - Medical | 0.69 (0.52-0.91) | 0.76 (0.57-1.01) | 0.061 |
| - Surgical | 1.07 (0.81-1.39) | 1.09 (0.83-1.43) | 0.541 |
|  |  |  |  |
| Clavien-Dindo grade ≥III | 0.79 (0.55-1.14) | 0.85 (0.59-1.23) | 0.401 |
|  |  |  |  |
| Postoperative ICU | 0.72 (0.54-0.96) | 0.82 (0.61-1.10) | 0.178 |
|  |  |  |  |
| Advanced stage (IIb+) | 0.56 (0.45-0.70) | 0.58 (0.46-0.72) | <0.001 |
|  |  |  |  |
| AJCC stage 4, % (n) ^C^ | 0.63 (0.43-0.93) | 0.66 (0.45-0.97) | 0.035 |
|  |  |  |  |
| R0 resection ^E^ | 2.46 (1.21-4.99) | 2.11 (1.04-4.30) | 0.039 |
|  |  |  |  |
|  | **Crude Reg. Coeff.**  **(95% CI)** | **Adj. Reg. Coeff.**  **(95% CI)** | **Adj. p ^A^** |
|  |  |  |  |
| Length of stay in days | -1.8 (-2.7; -0.8) | -1.5 (-2.4; -0.5) | 0.002 |
|  |  |  |  |

OR: Odds ratio; CI: Confidence Interval; Adj: Adjusted; Reg.: Regression; Coeff.: Coefficient.

ICU: Intensive care unit. Advanced stage IIb+: T4+, N+, M+; AJCC: American Joint Committee on Cancer;

^A^ Logistic model for categorical outcomes; multiple regression models for continuous outcomes. All models have been adjusted for age, gender, BMI, ASA group, cancer location (rectum or not), multiple comorbidities and drugs.

^C^ Because of missing data, the model included only 10,202 observations.

^E^ Because of missing data, the model included only 10,235 observations.
